# Unlocking the potential of polymeric desalination membranes by understanding molecular-level interactions and transport mechanisms

**DOI:** 10.1039/d2sc04920a

**Published:** 2022-12-13

**Authors:** Trisha R. Nickerson, Emma N. Antonio, Dylan P. McNally, Michael F. Toney, Chunmei Ban, Anthony P. Straub

**Affiliations:** a Department of Chemical and Biological Engineering, University of Colorado Boulder Boulder CO 80309 USA michael.toney@colorado.edu; b Materials Science and Engineering Program, University of Colorado Boulder Boulder CO 80309 USA chunmei.ban@colorado.edu; c Renewable and Sustainable Energy Institute, University of Colorado Boulder Boulder CO 80309 USA; d Department of Mechanical Engineering, University of Colorado Boulder Boulder CO 80309 USA; e Department of Civil, Environmental and Architectural Engineering, University of Colorado Boulder Boulder Colorado 80309 USA anthony.straub@colorado.edu

## Abstract

Polyamide reverse osmosis (PA-RO) membranes achieve remarkably high water permeability and salt rejection, making them a key technology for addressing water shortages through processes including seawater desalination and wastewater reuse. However, current state-of-the-art membranes suffer from challenges related to inadequate selectivity, fouling, and a poor ability of existing models to predict performance. In this Perspective, we assert that a molecular understanding of the mechanisms that govern selectivity and transport of PA-RO and other polymer membranes is crucial to both guide future membrane development efforts and improve the predictive capability of transport models. We summarize the current understanding of ion, water, and polymer interactions in PA-RO membranes, drawing insights from nanofiltration and ion exchange membranes. Building on this knowledge, we explore how these interactions impact the transport properties of membranes, highlighting assumptions of transport models that warrant further investigation to improve predictive capabilities and elucidate underlying transport mechanisms. We then underscore recent advances in *in situ* characterization techniques that allow for direct measurements of previously difficult-to-obtain information on hydrated polymer membrane properties, hydrated ion properties, and ion–water–membrane interactions as well as powerful computational and electrochemical methods that facilitate systematic studies of transport phenomena.

## Abstract 2

We highlight molecular-level interactions that govern the performance of polymeric desalination membranes. We emphasize how these interactions affect transport and highlight emerging methods that allow for direct measurement of polymer properties.

## Graphical abstract

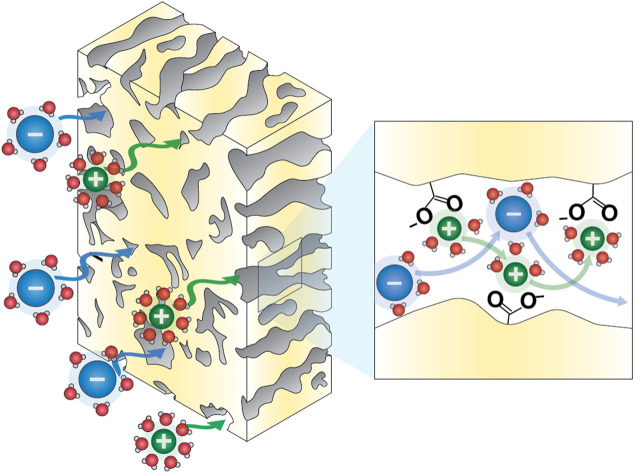

## 1 Introduction

Climate change, population growth, and industrialization are quickly reducing the availability and quality of fresh water supplies.^1^ As early as 2050, water scarcity threatens to be a reality for nearly 50 percent of the global population for at least one month per year.^2^ As climate change worsens, historically marginalized populations lacking geographic mobility, buying power, or political voice will be disproportionately affected by water insecurity, reinforcing social, economic, and health disparities.^3^

As conventional fresh water resources become less reliable, technologies to generate water from previously unusable sources such as seawater and wastewater will be required. Currently, reverse osmosis (RO) is the most widely implemented technology to produce fresh water outside of the natural hydrological cycle *via* desalination and wastewater reuse.^4,5^ RO is the process of driving water across a semipermeable membrane against its concentration gradient using an applied pressure. The concepts underlying RO were first discovered in the mid-1700s; however, it was not until the mid-1900s that the process was made energetically viable for commercial desalination with the development of high flux cellulose acetate membranes.^6^ In the 1970s, thin-film composite (TFC) polyamide (PA) membranes were created, achieving remarkably high water flux and salt rejection while being more durable than cellulose acetate in variable operating conditions.^6^

Although PA-RO membranes are widely implemented due to their excellent separation performance, they also suffer from long-standing problems that hinder performance^7–9^ and lead to premature membrane failure. Current PA-RO membranes show inadequate removal of certain compounds, such as low molecular weight neutral solutes, and have a limited ability to achieve solute–solute selectivity. The membranes are susceptible to scaling, fouling, and oxidative degradation from disinfectants.^10,11^ Computationally simulating membrane performance is also challenging since existing transport models cannot accurately predict performance under varied conditions.

It is difficult to address the limitations of PA-RO membranes with our current molecular-scale understanding of ion/water transport through membranes.^12^ Firstly, there is a poor understanding of the hydrated structure of PA due to the extremely thin and heterogeneous nature of PA membranes and the lack of *in situ* (hydrated, high pressure) characterization. Further, our understanding of the mechanisms that control ion transport is insufficient due to the difficulty of probing ion–polymer interactions in realistic environments. The availability of powerful experimental, computational, and characterization techniques has grown in recent years, presenting an opportunity to vastly improve our understanding of the mechanisms underlying the impressive performance of PA-RO membranes and to overcome remaining challenges.

The aim of this Perspective is to explore the nanoscale interactions within a PA-RO membrane by considering how ion, membrane, and solution properties affect ion–water–membrane interactions and how these in turn impact the transport properties of the membrane. The effect of nanoscale interactions is currently simplified into the phenomenological, continuum descriptions that are used to explain membrane performance. By deepening our understanding of these molecular level interactions, we can better tune them to control transport behaviour. Nanoscale interactions serve as a bridge between membrane materials properties (chemistry and structure) and performance, as these interactions are directly linked to the ion and membrane properties and determine transport. If we can elucidate how intrinsic materials properties dictate interactions and how these interactions result in transport, then we can advance beyond the empirical, phenomenological descriptions used in transport models to obtain a deeper understanding that enables prediction of transport and control of membrane performance.

Beyond improving our ability to obtain clean water, a fundamental understanding of ion interactions with soft matter is relevant to polymer membranes for pharmaceutical and industrial separations, battery and fuel cell membranes, materials for pollutant cleanup, greenhouse gas capture technologies, and targeted therapeutics. We can only realize the versatility of membranes for advanced separations, energy, and medicine if we can leverage and control interactions to dictate performance.

**Fig. 1:**
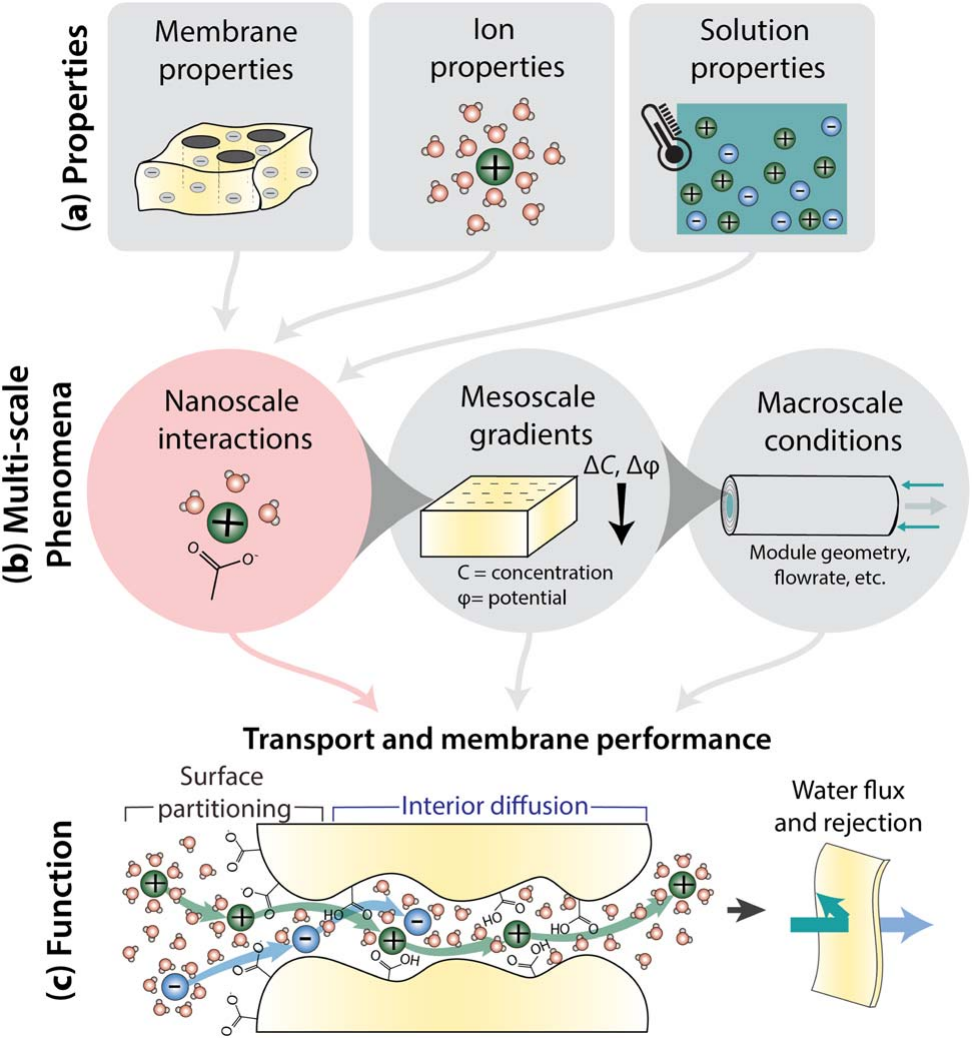
(a) Summary of the properties that give rise to interactions and govern membrane transport and performance: membrane properties such as morphology and chemistry; ion properties such as size, shape, and charge; and solution properties like composition, temperature, pH, and pressure. (b) Phenomena at many scales govern transport. Nanoscale interactions occur between ions and polymer groups while mesoscale gradients develop across the membrane thickness. Macroscale conditions like module geometry and flow parameters also affect interactions/gradients. Specifically, nanoscale interactions provide insight into the molecular level mechanisms behind (c) membrane function including surface ion partitioning and ion diffusion through the membrane interior. These transport phenomena dictate membrane permeability and selectivity performance.

Fig. 1 illustrates how nanoscale interactions relate membrane properties to performance. Important membrane properties include porosity, morphology and polymer chemistry, while ion properties include size, shape, charge, and hydration. Solution properties include composition, temperature, pH, and pressure (Fig. 1a). Phenomena over multiple length scales govern the performance of full-scale membrane modules (Fig. 1b). On the nanometer length scale of ions, water molecules, and membrane functional groups, ions and the membrane become hydrated and there are local bonding interactions. As we zoom out to the scale of membrane thickness, these nanoscale interactions occur within the context of mesoscale gradients in concentration and potential that drive mass transfer in the system. Finally, on the length-scale of membrane modules, the forces created by interactions and gradients intersect with macroscale factors, like module geometry and flow patterns, to determine overall transport. In this paper, we explore the relationship between nanoscale interactions and transport. Ion transport will be discussed in terms of surface and interior regimes, as illustrated in Fig. 1c since properties and interactions differ in these regions.^13^ Ion transport from the bulk feed solution, through the interface, and into the membrane is referred to as partitioning while diffusion is ion transport through the membrane interior. Transport behaviour in surface and interior regimes can be used to understand overall membrane performance, typically evaluated by water permeability and salt selectivity metrics, where both high permeability and high selectivity, or high “permselectivity”, are desirable.^14–16^

This Perspective is divided into sections which span increasing length scales from the intrinsic properties of the membrane and ionic solution to nanoscale interactions to measureable performance. We first explore fundamental properties of ions, membranes, and solutions (Section 2). We then identify nanoscale interactions that we expect in PA membranes and consider how they relate to transport (Section 3). These nanoscale interactions are then connected to established transport models and we highlight knowledge gaps that hinder the development of better technologies (Section 4). Finally, we offer a perspective on the use of state-of-the-art experimental and computational techniques to fill knowledge gaps to enable the discovery and development of improved membranes that support equitable access to high quality water (Section 5 and 6). Throughout this paper, we will draw upon theory and experimental data from research on membranes, polymers, and related materials to better understand interactions in RO membranes while highlighting the connection between technical challenges in these fields.

## 2 Properties

In this section, we will explore the properties, or physical and chemical characteristics, of the constituents of the ion–water–membrane system. These properties give rise to the various nanoscale interactions that will be discussed in subsequent sections.

### 2.1 Hydrated membrane

**Fig. 2:**
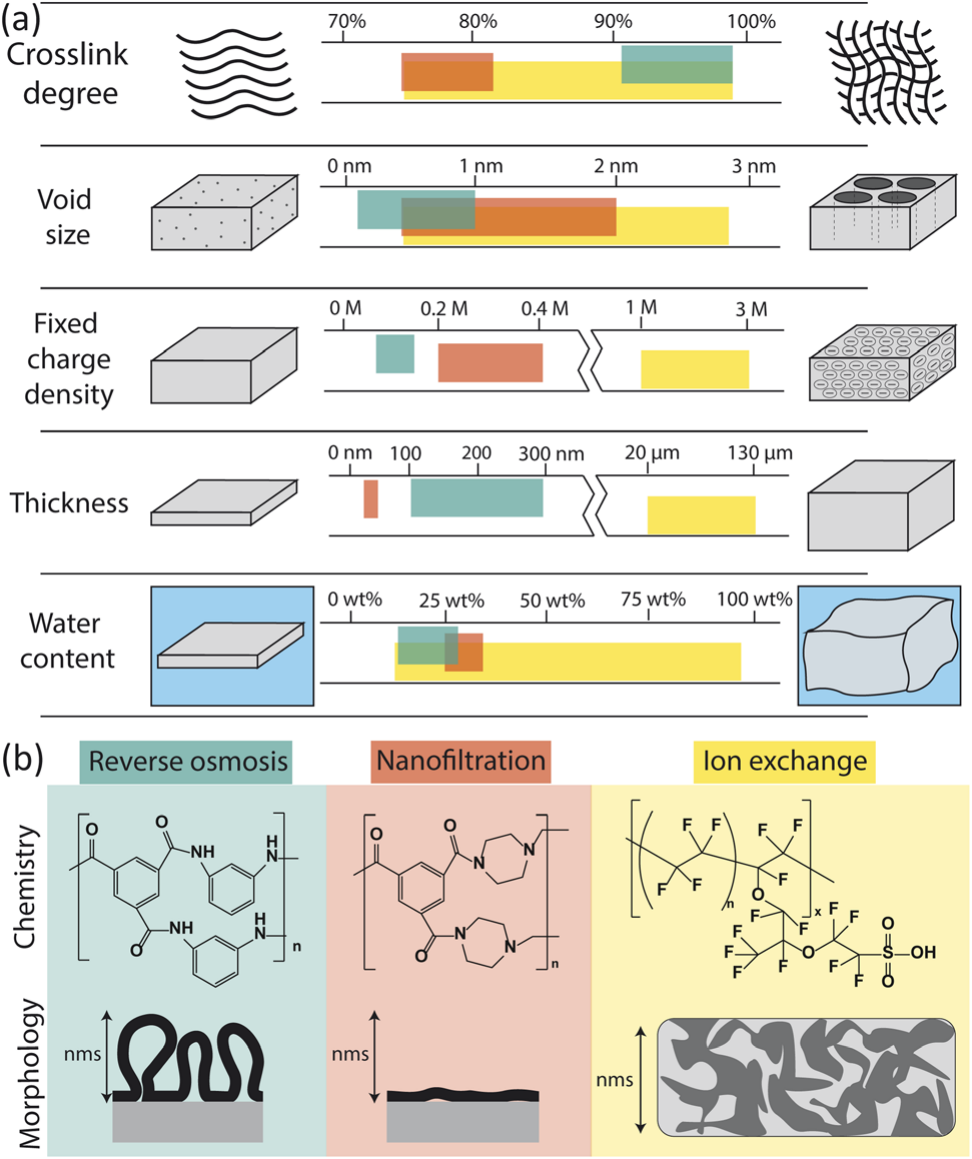
(a) Comparison of membrane properties for reverse osmosis (RO), nanofiltration (NF), and ion exchange (IX) membranes.^23–33^ Units for fixed charge density are moles of charge per liter of free water in the membrane. (b) Chemistry and morphology of RO, NF, and IX membranes. RO and NF chemistry is based on interfacial polymerization of trimesoyl chloride (TMC) and *m*-phenylenediamine (MPD) for RO or piperazine (PIP) for NF. Here only the fully crosslinked chemistry is shown; in actual membranes, unreacted portions lead to carboxylic acids, carboxylate, and amino groups in place of some amide groups. IX chemistry of a traditional Nafion membrane is shown.^34^ The morphology of RO, NF and IX membranes vary on the nm length scale. Adapted from ref. 35 and 36.

The physical and chemical properties of RO, nanofiltration (NF), and ion exchange (IX) membranes impact the way they interact with water molecules and ions, and thus determine their permselectivity performance. Fig. 2 summarizes the properties of each membrane type including chemistry, morphology, and other physical characteristics (crosslink density, void size, charge density, thickness, and water content). Here, we only consider the properties of hydrated membranes as relevant to their operation despite the extensive characterization of dry membranes in the literature.

Membrane properties are a result of reagent chemistry and synthesis conditions. A thin layer of PA, which performs the separation in RO and NF membranes, is typically synthesized *via* the interfacial polymerization of trimesoyl chloride and an amine monomer on top of a microporous polysulfone support. The chemistry and structure of the support layer has been shown to affect the roughness, hydrophilicity, crosslink density, and morphology of the PA layer.^17,18^ For RO membranes, *m*-phenylenediamine is used as the amino group, forming a highly crosslinked, fully aromatic film. For NF membranes, piperazine is generally used, resulting in a less crosslinked film.

The crosslinked portion of a PA membrane contains amide groups while unreacted regions contain carboxylate, carboxylic acid, and primary amino (RO) or secondary amino (NF) groups that give the membrane a slightly negative surface charge and hydrophilic properties.^13,19^ High crosslinking and high carboxylate density have both been shown to be essential for the selective performance of PA.^20^ Studies suggest that two p*K*_a_s exist for carboxyl groups depending on whether they exist at the membrane surface or in the interior.^13,21,22^ A p*K*_a_ at pH 5.5 is attributed to surface groups that deprotonate similarly to unconfined carboxylic acid groups. The second p*K*_a_ at pH 9.5 is thought to correspond to confined carboxyl groups that do not deprotonate as easily as a result of decreased dielectric constant or decreased pH in the membrane interior.

In addition to chemistry, membrane morphology impacts performance, but there is disagreement in literature about the structure of PA membranes and the existence of fixed (*i.e.*, static) pores or voids.^35,37–39^ With respect to transport models, RO membranes are generally considered to be nonporous or “dense” and transport is expressed by a single permeability coefficient and concentration gradient.^37^ At a molecular level, the “dense” membrane assumption implies that voids (really fluctuating free volume elements) develop and disappear as species diffuse through the voids. However, some work has argued that voids are “permanent”—or more accurately long-lived compared to diffusional timescales—describing these permanent voids as tunnels that continually swell and contract in response to external stimuli such as water activity and solution composition.^38^ Polymer chain dynamics play a key role in the fluctuation of free volume elements, but this area has lacked research attention. Herein, we refer to water-filled spaces between polymer chains as voids or pores to be consistent with past work, but this does not imply straight, cylindrical, or even long-lived pores.

RO membranes are generally characterized by their very high crosslink density, small void size (0.1–1 nm), low charge density, small thickness (100–300 nm), and low water sorption (Fig. 2a).^25,33,43,44^ Water uptake has been reported as low as 12–14 wt% based on quartz crystal microbalance (QCM) measurements^44,45^ but more commonly has been reported between 20 and 28 wt% based on weight gain upon hydration, gravimetric sorption analysis, and reports from commercial membrane manufacturers.^46–48^

Studies on the morphology of PA-RO membranes have identified a distinct ridge-and-valley structure with ballooning features suspected to originate from reaction–diffusion instabilities during interfacial polymerization (Fig. 2b).^35,49^ Culp *et al.* used electron tomography to determine that the peaks of ridged features on the PA are high density regions compared to the valleys of the film.^33^ These authors also generated three-dimensional density maps of PA to show that structural inhomogeneities create tortuous flow paths for water. It was found that a thick membrane with a uniform low density (above a critical density) is optimal for high permselectivity performance since water experiences a more linear flow path. In such membranes, the hydrophilic functional groups, highly crosslinked nature of PA, and smaller size of free volume elements lead to high water permeability and nearly complete removal of dissolved solutes, including monovalent ions.

NF membranes generally have lower crosslink density, larger void size (0.5–2 nm), higher charge density, and lower thickness (20–40 nm) than RO membranes.^23–28^ NF membranes tend to have a higher water content than RO membranes, with QCM measurements showing measured values of 25 wt%.^45^ However, additional water sorption measurements for NF membranes could not be found in the literature and, based on the relatively low water contents obtained by QCM compared to other techniques for RO membranes, we expect water sorption by NF membranes to be at least 25 wt%. NF morphology is similar to RO but films tend to be less crosslinked, thinner, and smoother, resulting in higher water permeabilities and lower salt rejections than RO membranes.

IX membranes are known for their high charge density, large thickness, and swelling behaviour.^29–32^ IX membranes are typically synthesized by solution casting and subsequent phase inversion, resulting in thicker, more porous polymers. The morphology of an IX membrane is heterogeneous with aggregated charged polymer domains and swollen solvent channels for ion transport.^36^ Although chemistries vary, a typical IX membrane, Nafion, consists of fluorinated carbon chains and a high density of sulfonic acid end groups.^50^ The highly charged, swollen nature of IX membranes allows them to selectively transport cations or anions in flow batteries, fuel cells, and electrodialysis for desalination.

Although we understand the general properties of desalination membranes, the exact nanoscale membrane structure and its relation to performance are still unclear. Typical measurements to characterize membrane properties include atomic force microscopy (AFM) for roughness and topography, contact angle for hydrophilicity, and X-ray photoelectron spectroscopy (XPS) for composition and crosslink density. It has proven challenging to evaluate local morphology and chemistry and their implications for performance using these techniques. For example, AFM imaging is unable to capture the structural complexity of the folded balloon-like structures that form the surface of the film since much of the topography is hidden by other features.^51^ Consequently, it is unclear if surface heterogeneity is advantageous for PA performance. Membrane roughness is thought to increase the surface area for water transport and fixed surface charge groups for ion rejection. However, surface roughness has also been linked to increased fouling and Culp *et al.* propose that the high mass-density of surface polyps make them “dead-regions” for transport.^33^

### 2.2 Hydrated ion

Ions interact with polymers and transport through membranes differently depending on their physical and chemical properties. Here we focus on ion properties like size, charge and hydration as they relate to ion interactions in solution and ion selectivity by the membrane. Ions are surrounded by solvation shells of various strength, size, and even shape that influence their subsequent interactions and transport. Ionic radii vary significantly depending on how ion size is determined. Bare ion radius represents the ion without an associated hydration shell, while hydrated ion size includes the oriented water molecules surrounding an ion. Another common representation of ion size is the empirically determined Stokes–Einstein radius that calculates the size of a hard sphere that diffuses at the same rate as the ion and is based on ion diffusivity and solvent viscosity.^52^

Table 1 illustrates the relative importance of ion hydration for various ions by comparing their bare, hydrated, and Stokes–Einstein radius and Born solvation energy.^40–42^ A highly charged ion with a small ionic radius, like Mg^2+^, can have a large hydrated radius, strong solvation energy, and the diffusion behaviour of a much larger ion according to its Stokes–Einstein radius. Conversely, a weakly charged ion with a large ionic radius, like K^+^, has the diffusion behaviour of an ion more similar in size to its unhydrated state due to its weak solvation shell. Regardless of the method of ion radius determination, ions are typically treated as hard, spherical, non-interacting particles in transport expressions.^53^ However, hydration structures not only influence the steric hindrance of an ion by increasing its size but also screen its charge, affecting subsequent interactions with other ions and polymer functionalities. Furthermore, understanding the impact of solvation on interactions and transport requires a more nuanced picture of ion hydration, especially within the membrane.

**Table 1:** Radii and solvation free energies for common ions in seawater and brackish water. Ionic radii were determined by various methods.^40,41^ Hydrated radii were obtained from simulated and experimental radial distribution functions.^41,42^ Ionic and hydrated radii were averaged from different sources and given with their standard deviations (±) and compared to calculated Stokes–Einstein radii.^41^ Finally, Born free energies of solvation were obtained from the literature^42^

Ion	Ionic radius (Å)	Hydrated radius (Å)	Stokes–Einstein radius (Å)	Born solvation energy (kcal mol^−1^)
Na^+^	1.0 ± 0.4	3.0 ± 0.9	1.8	−87.2
K^+^	1.4 ± 0.1	3.1 ± 0.4	1.3	−70.5
Mg^2+^	0.7 ± 0.1	3.2 ± 1.6	3.5	−437.4
Ca^2+^	1.0 ± 0.1	3.2 ± 1.2	3.1	−359.7
Cl^−^	1.8 ± 0.1	2.8 ± 0.8	1.2	−81.3
SO_4_^2−^	2.3	3.3 ± 0.1	2.3	−258.1

**Fig. 3:**
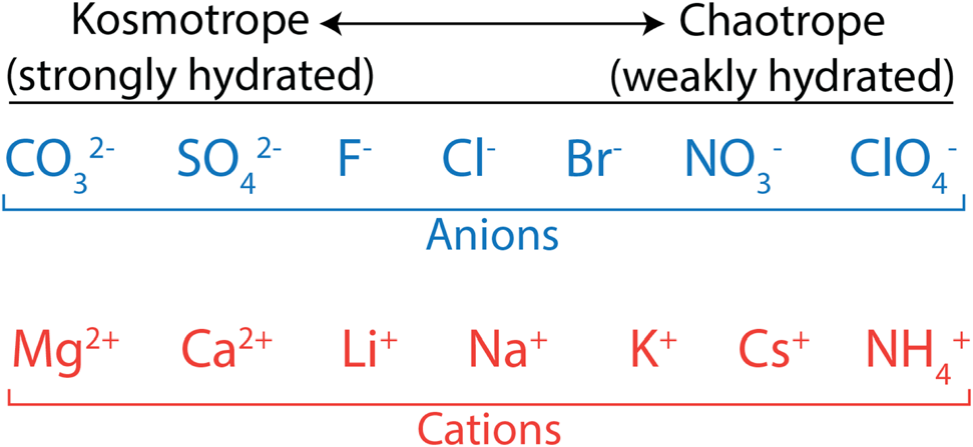
Kosmotrope to chaotrope classifications for anions (top) and cations (bottom). These are ordered in accordance with the Hofmeister series.

Biologists have extensively studied ion properties and interactions with biomolecules, and these insights can be leveraged to better understand the behaviour of ions as they relate to polymer membranes. Ions and other charged groups are split into two empirical categories based on their intrinsic properties and their tendency to interact with different materials.^54^ Small, strongly hydrated ions are known as kosmotropes. These ions, also called “structure makers”, break up the hydrogen bond network in water in favour of electrostatically-induced orientation of water around the ion. Kosmotropes also interact strongly with charged groups and surfaces. On the other hand, chaotropes are large, weakly hydrated ions, also called “structure breakers”, which have weak interactions with water and other polar species. In general, chaotropic ions have sizes more similar to their unhydrated state in solution, while kosmotropes behave like larger ions and move with their hydration shells intact. This is illustrated in Fig. 3 that shows how typical anions and cations are ranked from kosmotropes to chaotropes. We note that this ranking follows the Hofmeister series, dating back to 1888,^55^ and used to describe ion-specific trends in biology.^54,56–58^

Recent studies suggest that beyond hydration, ion size, charge, and shape (molecular ions) are important to ion–polymer interactions and transport, warranting further investigation.^59^ Work on PA and IX membranes has generally found that transport behaviour is dominated by ion hydration strength.^60–62^ However, these studies have tested a limited range of ions and experimental conditions such as pH and concentration that affect ion-specific phenomena in biological systems.^40,54^ A recent paper by Ritt *et al.*, studying cellulose acetate membranes and monovalent sodium salts, showed that ion hydration energy was only weakly correlated to the overall ion free energy and the resulting permeability–selectivity performance of the membrane.^13^ Entropic considerations and electrostatic interactions were reported to be more influential to overall performance than hydration.

### 2.3 Solution

Operating conditions can alter the properties of ions and membranes and affect subsequent ion–water–membrane interactions. While membrane and ion properties can be studied independently, consideration of solution properties—such as composition, solvent, temperature, pH, and pressure—is important as membranes encounter a wide range of feed solutions.

Even in the ideal case of a single salt solution, concentration acutely impacts rejection. High concentrations significantly increase salt permeability which is attributed to ions saturating membrane fixed charges and reducing the membrane charge density and the subsequent repulsion of ions.^63^ High ion concentration can also increase cation–anion interactions through ion pairing within the membrane.^64^ Low ion concentrations may result in complicated ion interactions with the membrane despite the common assumption that dilute solutions behave ideally.^53^ However, polyelectrolyte studies have shown that ions do not behave ideally at low concentrations, and IX studies have found poorer agreement between models and experiments at low concentrations.^65–67^ The non-ideal behaviour observed in dilute solutions could be explained by strong ion–polymer interactions and ion complexation with multiple functional groups due to reduced charge screening of membrane functional groups by other ions.^65^

Realistic feed waters contain a mixture of different solutes,^68^ resulting in interactions between solutes and competition for membrane functionalities. In complex feed waters, there is a finite concentration of membrane functional groups that hydrogen bond with water molecules and/or interact electrostatically with the ions. Interactions between ions and functional groups depend on membrane charge and membrane hydration which will vary depending on the amount of ions and water molecules that screen charged groups.

Higher temperatures increase water and ion permeability, which has been attributed to decreased solution and polymer viscosity.^69^ The increase in ion diffusivity is likely due to higher thermal kinetic energy of ions that enables desolvation and reduces transport resistances caused by attractive interactions with polymer groups. A recent study found that small, strongly hydrated Li^+^ had a lower permeability through PA than larger ions, K^+^ and Cs^+^, at low temperatures but a higher permeability at elevated temperatures when dehydration is more facile. This suggests that ion dehydration can be a rate limiting step to ion transport but this likely depends on other operating conditions.^62^ Higher temperatures also result in increased motion of polymer chains that increase the void dynamics during transport, increasing both water and ion permeation. However, the relationship between polymer chain dynamics, the nature of voids, and transport remains unknown.

pH determines the extent of dissociation of functional groups, membrane charge, and ion speciation in solution.^70^ The protonation of functional groups at the membrane surface and in the interior determines the membrane charge and whether hydrophobic or electrostatic forces dominate.^21^ In PA-RO membranes, increased pH has been shown to significantly increase salt rejection due to stronger electrostatic repulsive forces between anions and negatively-charged membrane functionalities.^13^

Applied hydraulic pressure results in membrane compaction while providing a driving force for water and solute transport. There is some evidence that pressure increases salt permeability despite higher transport resistance from membrane compaction.^63,71^ Pavluchkov *et al.*, compared ion permeability through membranes with and without applied pressure.^62^ In unpressurized diffusion experiments, it was shown that ion permeation increased with decreasing *hydrated* ion radius. When pressure was applied, ion permeability increased three-fold and the cation selectivity order was reversed: the ion permeation increased with decreasing *dehydrated* ion radius. This suggests that pressure-assisted ion dehydration occurs.

Although this Perspective focuses on water, the solvent has a significant influence on how ions and polymers behave in solution. The polarizability of a solvent influences the solvation shell size of an ion and the solvation of the polymer. Differences in permittivity and polarizability between the solvent and membrane surface determine where ions favourably accumulate. For example, in solvents with relatively low polarizability like water, polarizable ions have a stronger tendency for a charged surface.^54^

## 3 Interactions

In this section, we explore the nanoscale interactions that occur as ions and water molecules partition into and diffuse through the membrane. Consideration of ion, membrane, and solution properties discussed above will aid in understanding these interactions. Multiple interactions compete to determine the transport of ions and resulting membrane performance, which will be discussed in Section 4. In this section, we assume a negatively charged membrane so that the anion is the co-ion (same charge as membrane) and the cation is the counter-ion (opposite charge as membrane).

**Fig. 4:**
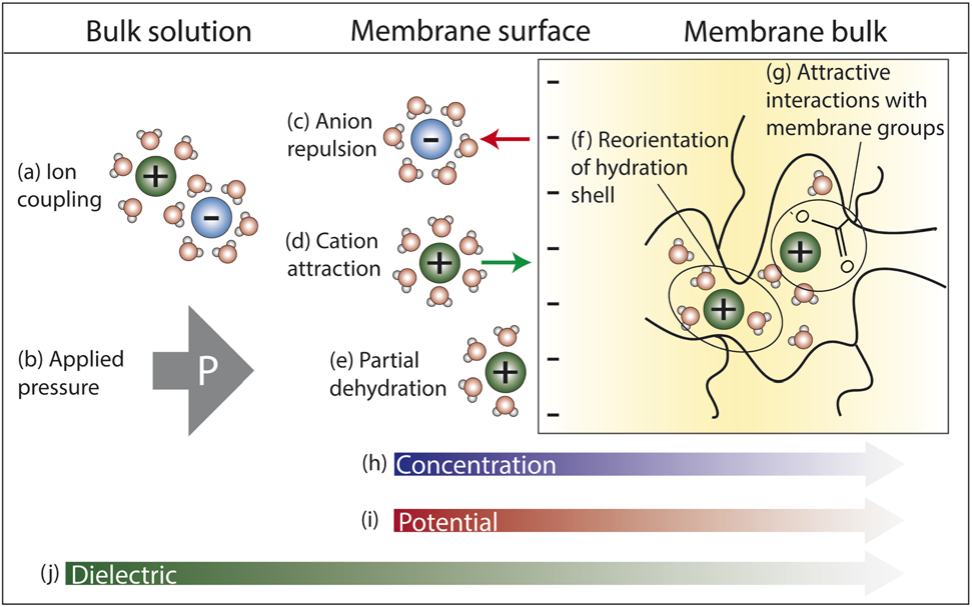
Interactions that an ion experiences as it moves from the feed solution into the (negatively charged) membrane. In the bulk solution, (a) dissociation of cation–anion pairs pose an energetic barrier to partitioning into the membrane, while (b) an applied hydraulic pressure is a driving force for water and ion transport. At the surface, (c) anion repulsion from the negatively charged membrane and (e) partial ion dehydration present energy barriers to membrane entry, while (d) attractive cation–membrane interactions partially offset these repulsive forces. In the membrane interior, (f) reorganization of an ion's hydration shell as it traverses the heterogeneous void network and (g) interactions with charged membrane groups present resistances to diffusive transport. Finally, the (h) concentration and (i) potential gradients created between the feed and permeate side of the membrane create driving forces for ion transport while a (j) dielectric gradient from the bulk solution through the membrane poses an additional transport energy barrier.

Fig. 4 illustrates the competing interactions and driving forces that an ion experiences as it moves out of the bulk feed solution, partitions into the membrane surface, and diffuses through the membrane interior. The membrane surface and interior have distinct properties that influence the hydration in each regime and cannot be described by average properties. Ion partitioning at the membrane surface is determined by a solute's local environment in the bulk solution *versus* in the membrane including association with other ions (Fig. 4a), hydration state (Fig. 4e), and attractive/repulsive interactions (Fig. 4c and d). Note that ion–polymer interactions may have an opposite effect on transport at the membrane surface compared to inside the membrane interior. For example, attractive cation–polymer interactions stabilize ions at the membrane surface (Fig. 4d), increasing ion partitioning, but create resistances as ions move through the membrane (Fig. 4g), slowing ion diffusion. Meanwhile, gradients across the membrane create additional driving forces to transport (Fig. 4b and h–j).

### 3.1 Water–membrane

The interactions between the polymer and water molecules in the membrane determine the dielectric and electrostatic environment that ions experience, affecting the ion–polymer interactions and transport in the system. Water in the membrane is currently described in terms of average properties such as water sorption, membrane degree of hydrophilicity, and average dielectric constant.^72^ However, this approach does not account for the known polymer heterogeneity. Here, we discuss the limitations of this averaging, using PA as an example, and outline a path towards a more realistic picture of water within the membrane and the fluctuating voids.

The membrane surface and interior have distinct properties that influence the hydration in each regime and cannot be described by average hydrophilicity and water content. Membrane swelling is highly dependent on functional groups and has been shown to vary between 15.7 and 32.2% depending on the ionization of amino and carboxyl groups in PA-RO membranes.^13^ Interestingly, water permeability was shown to increase non-linearly with swelling, as a twofold increase in swelling only resulted in a 10% permeability increase; this was attributed to increased swelling in the surface PA layers where there is a higher concentration of deprotonated functionalities compared to the membrane interior.^13^ The nanoconfinement of water molecules in the interior PA suppresses the deprotonation of functional groups, decreasing internal membrane charge and reducing internal swelling and consequent water transport.

**Fig. 5:**
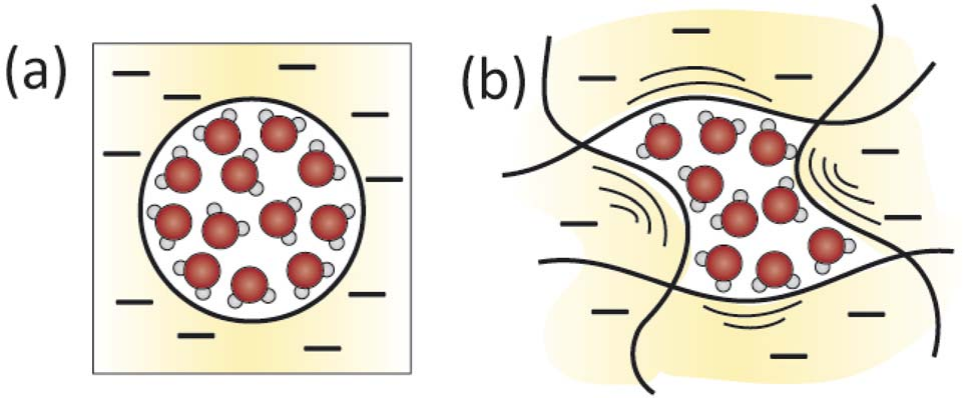
An illustration of the assumptions used to calculate the dielectric constant of water in the membrane. (a) Straight, cylindrical pores with one layer of oriented water molecules on pore walls are assumed and the remainder of water is assumed to behave like the bulk solution. The effective dielectric constant of the water in the pores is the weighted average of the dielectric constants of the water at the pore wall and interior. (b) A more realistic picture of water in the dynamically fluctuating voids where water molecule orientation spatially and temporally fluctuates.

Currently, the permittivity of the hydrated membrane is described by highly simplified models and it is essential to implement a more realistic description of the dielectric environment of hydrated membranes to accurately predict transport properties. The ability of water molecules to hydrogen bond with membrane functionalities and solutes depends on water's capacity to freely re-orient in the membrane, as described by the dielectric constant.^72^ This re-orientation is further influenced by the extent of confinement of water molecules in the membrane pores. Fig. 5a shows the typical model used to approximate the static dielectric permittivity of water within the membrane pores (an important property for determining the dielectric exclusion factor). Currently, an average dielectric constant is calculated based on the assumptions that there are uniform, cylindrical pores with one layer of water molecules oriented on the pore walls, while the remaining water molecules in the pore interior exhibit bulk-like behaviour.^53^ The present model in Fig. 5a makes many simplifying and unrealistic assumptions. For example, the assumption of bulk water in the pore interior may be inaccurate, since molecular dynamics simulations show that water has a linear hydrogen bond structure in nanoconfined PA compared to a tetrahedral structure in bulk solution.^73^ Therefore, it is imperative that we accurately determine the effective dielectric constant of the water in the membrane pores by accounting for more realistic morphological and chemical details of the PA, as illustrated in Fig. 5b, and by experimentally determining the dielectric constant of hydrated membranes.

A dielectric continuum is generally assumed between the solvent and polymer phases and ion sorption is determined by the free energy change associated with an ion partitioning from the feed solution into the membrane. The effective dielectric constant of the hydrated membrane can be obtained experimentally from that of the dry polymer and the water content of the hydrated polymer,^72^ assuming a linear variation of the dielectric constant with water content between the that of the dry polymer and the bulk solution.^53,74^ However, studies have shown that, in addition to water content, polymer chemistry (concentration and distribution of functional groups) and membrane structure have significant impacts on permittivity and should be included in realistic models.^65,75,76^

The state of water also influences its transport across the membrane, which in turn affects the hydration, dielectric, and electrostatic environment in the membrane. The combination of attractive interactions and confinement effects cause the self-diffusion coefficient of water to decrease through the membrane, dropping more than five times at the solution–membrane interface and twelve times in the membrane interior compared to the bulk water.^77^ Decreased water transport in some regions of the membrane as well as preferential accumulation of water around charged functionalities implies the dielectric constant likely varies throughout the heterogeneous membrane.^77^

### 3.2 Cation–anion

Interactions between cations and anions have implications on overall salt selectivity. Cation–anion pairing within membranes is generally assumed to be negligible because the ion concentrations in typical feedwaters are 1–35 g L^−1^ (ref. 78) and there is little ion pairing in water at these concentrations for monovalent ions.^64^ However, even at typical feed salt concentrations, ion–ion interactions may be facilitated by concentration polarization at the membrane surface, decreased dielectric constant within the membrane, and concentration of salt within the membrane. Ion association between divalent ions is prevalent in seawater,^64,66,79^ and ion pairing is even more likely in the lower dielectric environment of PA membranes, based on polyelectrolyte studies.^80,81^ One electrochemical study compared transport energy barriers of individual ions to those of entire salts to show that cations and anions traverse the membrane independently.^59^ However, this study only analyzed monovalent ions which are significantly less likely to interact than divalent ions and thus the general question of cation–anion association in membranes remains unanswered.

Besides explicit ion pairing, co-ion (anion) identity has been shown to impact the partitioning and diffusion behaviour of the associated counter-ion (cation). Recall that we assume the PA is negatively charged here. While we could not find any work on PA membranes, Luo *et al.* studied the ion concentration of various sodium salts in a polyether ether ketone (PEEK) IX polymer, which has aromatic rings and carbonyl groups similar to PA and is also negatively charged under relevant conditions.^82^ The study found that the concentration of Na^+^ in the polymer was highly dependent on anion identity; sorbed Na^+^ concentration was four–five times higher when associated with NO_3_^−^ compared to anions such as F^−^ or SO_4_^2−^. Sorption trends were explained based on competition between the strength of cation–membrane attraction, anion–membrane repulsion, and cation–anion attraction, as illustrated in Fig. 4. A small, divalent anion experiences strong repulsion from the PEEK surface and strong association with Na^+^, effectively pulling Na^+^ out of the polymer and reducing the concentration in the membrane. In contrast, a larger monovalent anion experiences weaker repulsion by PEEK and weaker association with Na^+^, allowing Na^+^'s attractive interactions with the PEEK to dominate, and resulting in a higher concentration in the membrane.^82^

### 3.3 Ion–membrane

Ion–membrane interactions have significant implications on ion transport and a better understanding these interactions presents the opportunity to elucidate transport mechanisms that govern membrane permselectivity. Here, we explore relevant ion–polymer interactions based on the ion, membrane, and solution properties discussed in Section 2, and informed by studies on similar materials. Note that the same interactions that promote desolvation and attract ions to partition through the feed–membrane interface create resistances that slow diffusion through the membrane interior.

This subsection is broken into what we term average and local interactions, sometimes referred to as nonspecific and specific.^83,84^ Here, we use average interactions to refer in a general sense to attractive and/or repulsive forces between ions and the membrane's average field (electric, concentration). Local refers to nanoscale interactions between ions and polymer functionalities. Irrespective of the localization of interactions, partial ion desolvation is an important consideration affecting interactions and transport. When attractive forces between ions and the membrane surface overcome the energy penalty for partial desolvation, ions adsorb and partition into the membrane.^85,86^ According to recent studies, ions shed 1–3 water molecules from their hydration shell when they partition into the membrane.^19,62^ The dehydration energy has been shown to have a strong influence on ion selectivity in NF membranes where diffusion is more facile for ions that are partially dehydrated. This contrasts to highly-crosslinked NF or RO membranes that have voids that are typically smaller than the dehydrated size of many ions.^62^

#### 3.3.1 Average interactions

**Fig. 6:**
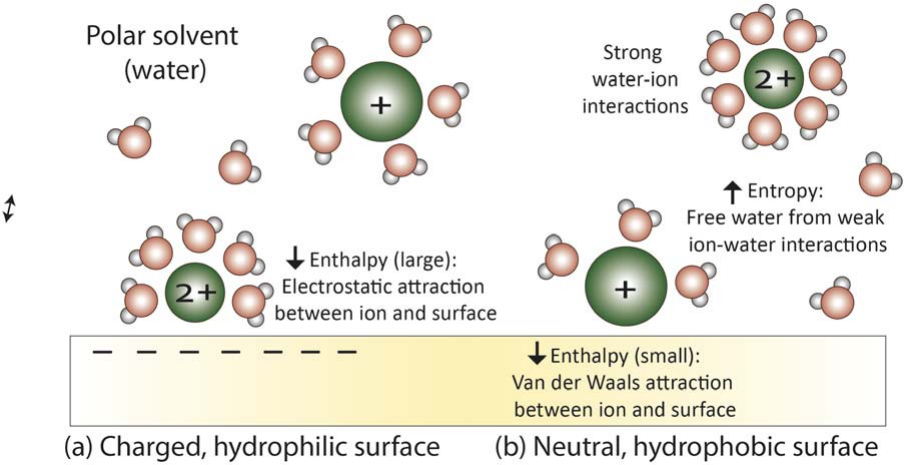
Average surface interactions at (a) a charged, hydrophilic surface where electrostatic forces dominate and attract small, strongly hydrated ions and (b) a neutral, hydrophobic surface where hydrophobic forces, a combination of entropic forces and van der Waals interactions, dominate to stabilize large, weakly hydrated ions at the surface.

In current transport models (Section 4), the membrane is treated as a continuum and interactions result from the attraction or repulsion of ions to the average membrane electrostatic field or through hydrophobic interactions. Fig. 6 shows average interactions that dominate at the surface of a (a) negatively charged, hydrophilic membrane or (b) neutral, hydrophobic membrane. When strong charges are present as in (a), small, high charge density ions (kosmotropes) accumulate near the charged surface due to enthalpically favorable electrostatic interactions.^54^ In the absence of strong charge effects as in (b), large, low charge density ions (chaotropes) are attracted to hydrophobic surfaces. These hydrophobic forces are a combination of favorable increases in entropy due to the liberation of water molecules when the ions become proximal to the surface and weak van der Waals interactions that enthalpically stabilize the ions near the surface.^83,87^ In this section, we focus on electrostatic interactions since PA is a charged, hydrophilic polymer.

The impact of electrostatic interactions on ion transport through RO membranes influences the empirically-determined partitioning and diffusion coefficients. In contrast, for NF and IX membranes, ionic charge and average membrane charge density are explicitly accounted for in transport models. Transport models are explained in more depth in Section 4, but we will briefly explore efforts to account for electrostatic interactions based on the ion exchange membrane literature.

Highly charged IX membranes have stronger electrostatic ion interactions than moderately charged NF/RO membranes, thus the effect of ion–membrane interactions on transport is more often considered for IX. In studies of IX membranes, counterion condensation theory (CCT) considers the tendency of ions to bind to polymer fixed charges, reducing the membrane's charge density. CCT is used to calculate ion activity coefficients to account for nonideal behaviour and modify transport predictions. In CCT, the polymer is treated as an infinitely long polyelectrolyte chain with fixed, equally spaced charged groups.^86^ Ion–polymer interactions are calculated from the linear charge density of the polymer and ion properties determined using one of three models: (a) Manning's theory, the simplest and most commonly used method, accounts for only ion charge, (b) electrostatic theory, based on the Born model, considers ion charge and hydrated ion size, and (c) the electrostatic and dispersion force theory includes ion charge density and excess ion polarizability.^65,86^

Attempts to describe an ion's nonideal behaviour using CCT have only been successful under certain conditions. Manning's theory was able to accurately predict ion sorption in the membrane phase for NaCl, but not at low concentrations when charge screening of fixed membrane groups was minimal.^65^ For more complex salts, NaBr and NaNO_3_, Manning's theory could not quantitatively predict sodium sorption values or qualitatively predict sorption trends for different salts at moderate external salt concentrations. Electrostatic theory was able to model sorption trends, but values were an order of magnitude too small.^86^ The electrostatic and dispersion theory had even less quantitative and qualitative agreement with experimental data than Manning's or electrostatic theory. These inaccurate predictions were attributed to a lack of ion- and membrane-specific parameters in the models and no consideration of ion–polymer interactions in activity coefficient calculations.^86^

#### 3.3.2 Local interactions

**Fig. 7:**
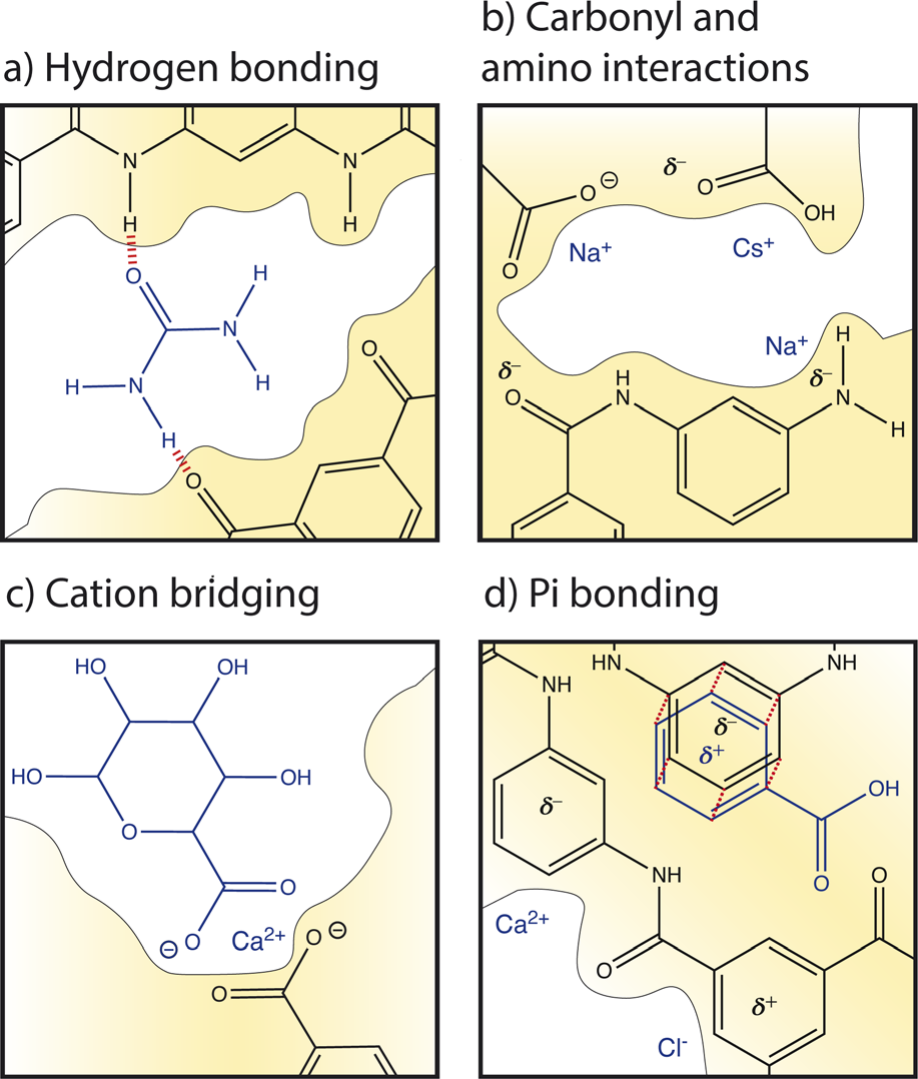
Summary of local nanoscale interactions between ions and membrane groups. (a) Hydrogen bonding occurs between hydrogens and electronegative atoms, illustrated with a urea compound. (b) Electrostatic interactions occur between ions and carboxylate, carboxylic acid, amide, and amino functional groups on the membrane. (c) Cation bridging occurs when a cation forms crosslinking bonds between carboxyl groups of organic matter and the membrane. Finally, (d) pi bonding occurs between aromatic rings on the membrane and cations, anions, and aromatic rings of organic matter.

Given the limited molecular knowledge of the hydrated PA membrane and ions within the membrane, only average interactions (and corrections for nonideal ion activities as in CCT) have been considered in data analysis and transport models. This average treatment ignores local ion–polymer interactions. A better understanding of these local interactions is important for elucidating realistic ion transport mechanisms which would provide critical information for improved membrane material design and development of predictive transport models. Fig. 7 illustrates some local interactions that may occur between ions and chemical functionalities in a PA membrane.

Several studies have recognized the significance of local cation–carboxyl interactions, illustrated in Fig. 7b, on membrane performance but the molecular details of these interactions are not well understood.^13,59,88,89^ Cation–carboxyl interactions are speculated based on the presence of carboxyl groups identified in dry PA membranes by X-ray photoelectron spectroscopy (XPS) and Fourier transform infrared spectroscopy (FTIR).^89–91^ Since the p*K*_a_ of a carboxyl group is below the neutral pH usually experienced in water treatment, carboxylic acids are assumed to deprotonate to carboxylates, resulting in PA's negative surface charge and repulsion of cations. However, as mentioned in Section 2, it is probable that only surface groups deprotonate due to nanoconfinement in the membrane interior, changing the p*K*_a_ to 9.5. The protonation of carboxyl groups is important for interactions and transport; one computational study varied pH to control the dissociation of functional groups to understand the trend of cation affinity to protonated carboxylic acid groups (COOH) and deprotonated carboxylate groups (COO^−^).^21^ At low pH, larger cations like Cs^+^ interacted more strongly with COOH groups than smaller cations such as Na^+^. In PA membranes, strong interactions with interior protonated COOH groups could be responsible for the slowed diffusion of Cs^+^ observed in an NF membrane.^62^ At high pH, carboxylic acids deprotonated to carboxylates and smaller cations with larger charge densities, like Na^+^ and Li^+^, preferentially interacted with these COO^−^ groups.^21^ Ritt *et al.* showed that as pH increased from 4 to 10 and the COO^−^ density of a commercial NF membrane increased, the rejection of NaNO_3_ increased by fourfold, indicating the significant role that carboxyl groups play in salt selectivity.^13^

Amino groups also contribute to PA's hydration properties and ion interactions (see Fig. 7b), albeit to a lesser extent than carboxyl groups due to their lower concentration and localization to the polymer–support interface.^13^ Unlike carboxyl groups, amino groups have a single p*K*_a_ at 9.5. Molecular dynamics simulations found that amino groups of PA-RO membranes maintain two hydrogen atoms in neutral conditions regardless of nanoconfinement and membrane dielectric constant.^13^ Furthermore, the amino density in the membrane interior is small and it is suspected that most amino groups are found at the interface between the PA and polysulfone support due to the conditions of the interfacial polymerization reaction. Thus, amino groups probably do not affect ion partitioning but hydrophilic amino groups can increase membrane hydration and may affect water and ion permeability.^13^

Cations can also interact with the oxygen atom of amide groups which are prevalent in the highly crosslinked PA network (see Fig. 7a). In a hydrated membrane, two water molecules hydrogen bond to the amide oxygen.^92^ Molecular dynamics show that when a Na^+^ ion approaches an amide O, one water is replaced but the hydrogen bonding network around the amide oxygen is not significantly altered. In contrast, a Ca^2+^ ion replaces both water molecules and binds to the amide oxygen.^92^

Pi interactions, shown in Fig. 7d, are rarely discussed in the membrane community and yet are likely significant to ion transport based on the high degree of aromaticity in PA-RO membranes. In contrast, pi interactions are commonly considered in organic electronics, soil interactions, and biological systems.^87,93,94^ Similarities in the degree of aromaticity, functional groups (amino, carboxyl), and surrounding solutions (ions, organic matter) suggest that these interactions are important to account for in polymer membranes. While the strength of an single ion–carboxylate bond is greater than that of an individual ion–pi bond, pi bonds may have a large collective impact on interactions and transport due to the high degree of aromaticity in PA. Pi bonding occurs when the pi orbital of an aromatic ring has a partial charge due to excess or deficient electron density. Benzene rings typically have a partial negative charge in their pi orbital and substituent groups attached to the ring can either increase the negative charge by contributing more electron density to the pi orbital (electron donating) or neutralize/reverse the charge by drawing excess electron density from the pi orbital (electron withdrawing). It is unclear how aromatic rings in PA-RO membranes behave since the oxygen atoms of carboxyl and amide groups are electron donating, while the nitrogen atoms of amino and amide groups are electron withdrawing.^95^

Cation–pi bonding, between a cation and an aromatic ring with an electron-rich pi orbital, has been heavily researched and is likely prevalent in PA-RO membranes. A computational study of benzene interactions with different cations revealed that the strength of cation–benzene complexes in an aqueous environment was highest for Li^+^ followed by K^+^, Na^+^, and Rb^+^. However, for sandwiched benzene–cation–benzene complexes, K^+^ interactions were favored over Li^+^ due to K^+^'s lower desolvation energy and Li^+^'s propensity to bind more strongly to a single benzene ring due to its large electrostatic attraction.^85^ Sandwiched benzene–ion–benzene interactions may be relevant for ion transport through the sub-nm voids of PA-RO membranes where ions transport between aromatic layers. Cation–pi interactions have binding strengths up to 8 kcal mol^−1^ even when cations are hydrated by three water molecules.^87^ These interactions are stronger than hydrogen bonds between water molecules, suggesting that there could be a strong cumulative effect of cation–pi interactions in aromatic PA membranes.

Anion–pi interactions have received much less attention from the scientific community, but they may also be significant. These interactions are generally neglected because: (i) anions are assumed to interact repulsively with electron-rich benzene rings; (ii) anions often have lower effective charge densities than cations, weakening electrostatic effects; and (iii) anions have relatively high desolvation energy given their size, as shown in Table 1, further decreasing electrostatic interaction strength.^96^ However, anion–pi interactions have been shown to be energetically favourable, as well. The pi orbital of aromatic rings can have a partial positive charge when electron withdrawing groups are present, allowing attractive interactions with anions. The solution environment can also induce or alter charges in the polymer network; for example, ion-induced polarization of a non-electron deficient ring by a cation on one face can allow the ring to interact with an anion on its opposite face.^96^

Ion–membrane interactions not only influence ion transport but also affect membrane properties such as membrane charge and hydration. A better understanding of the molecular details of hydrated ions, membrane functionalities, and their interactions presents an opportunity to explain observations that cannot be captured by current theories and models. Understanding the molecular interactions between ions and the polymer is important for predicting transport and for evaluating the state of the membrane since osmotic deswelling (reduced water content in the membrane) has been shown to occur in saline solutions and affect membrane performance.^72^ We were unable to find studies on this topic for PA membranes but one study on the crosslinked copolymer poly(glycidyl methacrylate) found that an increase in NaCl concentration caused osmotic deswelling, or a decrease in the bulk-like water content in the membrane, and increased ion sorption in the membrane.^72^ Another study showed that when a negatively charged poly(sodium 4-styrenesulfonate) membrane was submerged in ionic solution, its water content increased with increasing strength of the cation's hydration shell.^97^

### 3.4 Organic matter

In addition to ion, water, and polymer interactions, organic matter can interact with the membrane and other solutes. Although fouling is not a focus of this Perspective, interactions with organic matter can alter ion and membrane behaviour and are important for predicting membrane performance and lifetime.

Electrostatic interactions can manifest between cations and the negatively charged groups of adjacent organic/biological molecules to form a crosslinking network, known as cation bridging, illustrated in Fig. 7c. Cation bridging can significantly increase the permeation resistance for water transport and can cause severe membrane fouling and performance degradation. These interactions are strongest for highly charged ions with weak hydration shells.^98,99^ Specifically, cation-bridging interactions occur most frequently between Ca^2+^ ions and carboxylate groups of natural organic matter (NOM), causing aggregation and charge neutralization. At high calcium concentration, Ca–NOM bridging results in thick, dense, gel-like layers due to calcium's weak hydration that allows calcium to dehydrate and form ionic bonds with NOM.^98^ Detrimental bridging interactions have also been observed with high Mg^2+^ concentrations.^98–100^

Deprotonated carboxylate groups on the membrane surface likely also engage in cation bridging interactions such as those that aggregate organic matter. Cation bridging between ions, NOM, and the membrane may cause “sticking” of aggregated foulants to the membrane surface. Bridging between organic matter and the membrane can neutralize membrane charge groups and reduce membrane surface area, impacting ion/water transport and exacerbating the severity and cleanability of membrane fouling.^99^

Organic matter in feed water can also interact with the membrane directly. There are some disagreement in the literature regarding the significance of different organic–membrane interactions. Molecular docking simulations have shown that hydrophobic forces dominate interactions with apolar organics while electrostatics govern interactions with charged organics.^84^ One study showed that local pi–pi and hydrogen bonding interactions, rather than average hydrophobic and electrostatic interactions, govern adsorption and subsequent partitioning into and diffusion through a PA membrane.^83^ It is likely that hydrogen bonding, illustrated between urea and PA functionalities in Fig. 7a, pi–pi bonding (Fig. 7d), and cation bridging interactions (Fig. 7c) are prevalent in the aromatic and functionalized PA environment. Due to the relatively large size and aggregation tendency of NOM, these organic–membrane interactions significantly disrupt membrane properties and ion/water transport behaviour.

## 4 Transport

In this section, we briefly review two common models for predicting water and salt permeation in pressure-driven membranes and relate these to the molecular-level properties and interactions discussed in previous sections. After reviewing current models, we discuss the assumptions that limit predictive capabilities. We note that this section specifically focuses on solution–diffusion and pore flow models since they are widely used, and the limitations of these models are broadly representative of challenges associated with modeling water and ion transport.

### 4.1 Solution–diffusion model

**Fig. 8:**
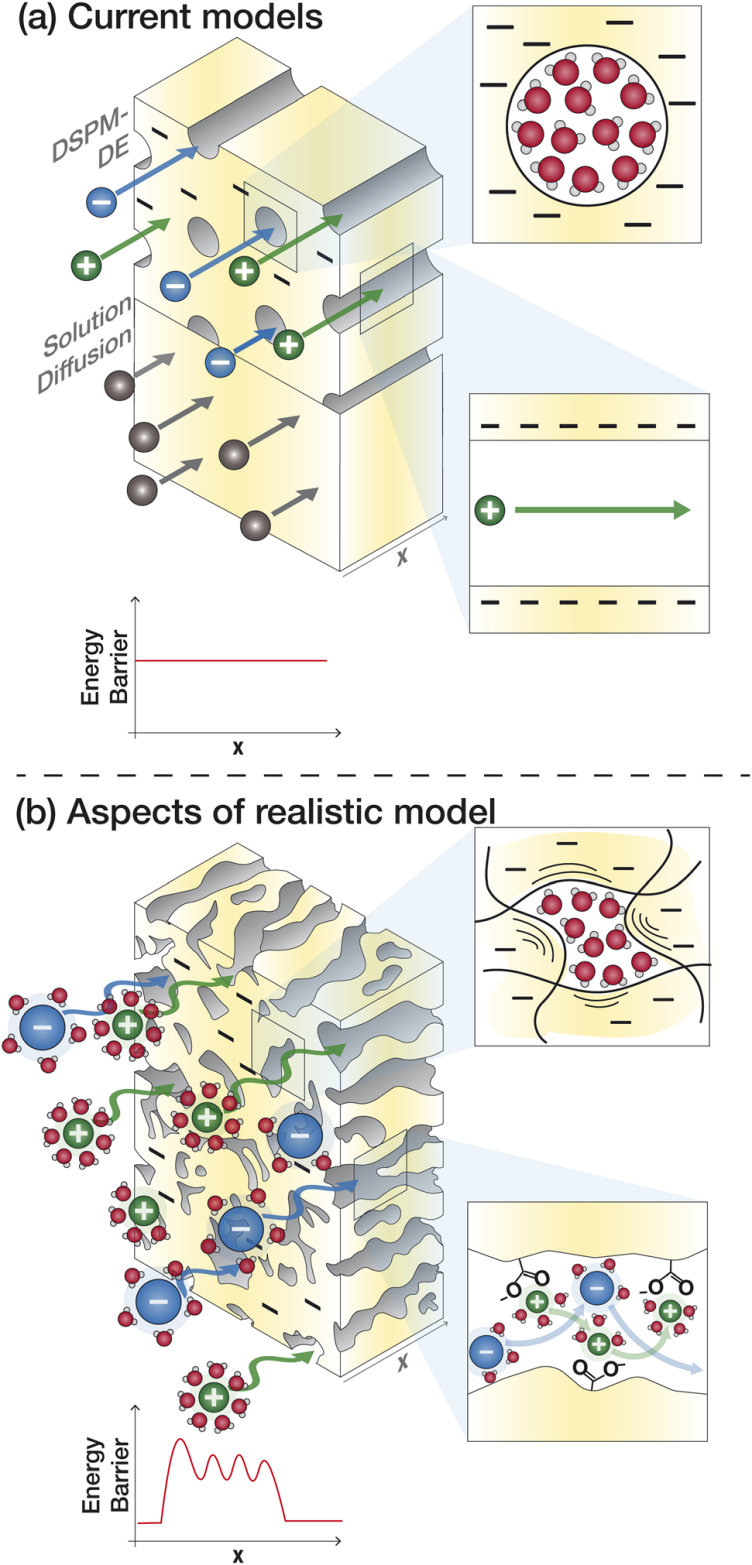
(a) Illustration of the treatment of ion transport by the Donnan steric pore model with dielectric exclusion (top) and solution–diffusion model (bottom) compared to (b) a more realistic model of ion transport with more accurately determined parameters for ion size, void size distribution, and dielectric constant that accounts for interactions and resistances that an ion experiences at the surface and interior regions of the membrane.

The solution–diffusion (SD) model is the dominant model used to calculate salt and water flux in RO membranes and is illustrated in Fig. 8a. It provides a useful framework to conceptualize the transport of water and solutes based on partitioning into and diffusion through a membrane down a chemical potential gradient. The membrane is considered uniform and nonporous. The polymer and sorbed solvent are treated as a single phase, distinct from the bulk solvent feed. The SD model assumes a uniform high pressure across the entire membrane thickness and chemical equilibrium at the membrane interface.^37,101,102^

In the SD model, the flux of species i, *J*_i_, is defined as directly dependent on the chemical potential gradient, d*μ*_i_/d*x*, and a proportionality factor that relates driving force to flux, *L*_i_:^37,101,102^1
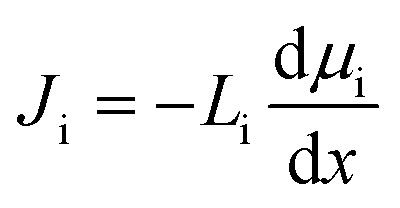


Since the pressure is assumed to be constant across the membrane, the chemical potential gradient of a species in the membrane is a function of the concentration gradient in the membrane phase according to Fick's first law:2
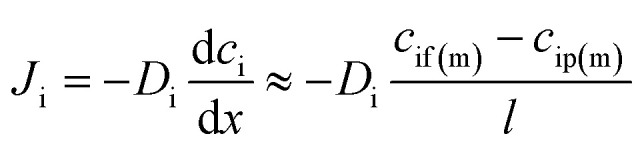
where *D*_i_ is the diffusion coefficient, *c*_i_(*x*) is the concentration, and *l* is the membrane thickness. *c*_if(m)_ and *c*_ip(m)_ are the concentrations of species i at the feed–membrane and permeate–membrane interfaces, respectively, which are related to the concentrations in the solvent phase assuming chemical equilibrium:3
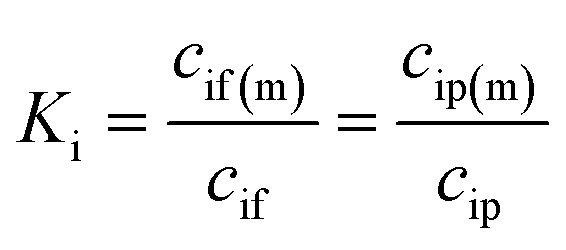
where *K*_i_ is the partitioning coefficient. *c*_if_ and *c*_ip_ are the concentrations in the solvent phase on the feed and permeate sides of the membrane, respectively. Combining eqn (2) and (3) yields the following expression for flux across the membrane.4
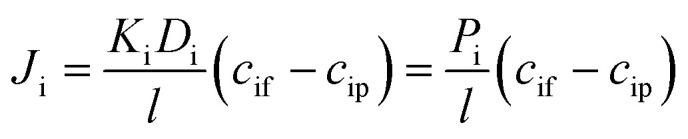


The phenomenological, empirically determined permeability coefficient, *P*_i_, is the product of the partitioning, *K*_i_, and diffusion, *D*_i_, coefficients. Eqn (4) is used to describe the solute flux across the membrane in RO systems in the SD model.

It should be noted that the concentration gradient (*i.e.*, the driving force) that an ion experiences is determined by the concentration of ions at the feed–membrane interface. Increased ion concentrations occur at the this interface due to a buildup of rejected ions at the membrane surface. Generally, the concentration at the membrane interface is estimated using a concentration polarization equation which is explained in detail elsewhere.^103^

The SD model is commonly used to explain solute transport with a single empirically determined parameter, the permeability coefficient (*P*_i_). Trends have been established for how the permeability coefficient of a solute varies with concentration, hydrated size, and charge.^11,104^ However, the use of the single parameter greatly limits the ability of the SD model to predict performance in varied conditions, complex feed waters, and when novel solutes are present because the predictions rely on fitted, phenomenological parameters that lack molecular details. Without an ability to incorporate a more detailed molecular-level understanding of the fundamental factors that govern transport, it is impossible to predict the impact of varied membrane properties and experimental conditions on performance. This is a major limitation of the SD model.

### 4.2 Donnan steric pore model with dielectric exclusion

A more complex model, the Donnan steric pore model with dielectric exclusion (DSPM-DE), was developed following proof of long-lived pores in NF membranes which required consideration of transport pathways beyond diffusion.^53^ The DSPM-DE is illustrated in Fig. 8a and is used to calculate flux through NF and IX membranes and, more recently, has been considered for RO membranes. The DSPM-DE uses eqn (5)–(7) to first calculate the concentration of ions that partition into the membrane based on size, charge, and solvation. The calculated surface concentration defines a boundary condition which is used to calculate ion flux through the membrane interior using an extended Nernst–Planck equation.^53^

In the DSPM-DE, the feed concentration that develops at the membrane surface is determined by accounting for ion exclusion at the surface through (i) steric, or size-based, rejection when ions do not fit through membrane voids, (ii) Donnan, or charge-based, rejection when anions are repelled by the negatively charged membrane, and (iii) dielectric exclusion when the restricted mobility of water molecules in the membrane results in a solvation energy barrier for ions. Exclusion factors are solved simultaneously assuming electroneutrality to determine the concentration of ions at the membrane surface which sets a boundary condition to calculate flux through the membrane.^53^

The steric, Donnan, and dielectric exclusion factors in the DSPM-DE model are defined in eqn (5)–(7), respectively. Steric exclusion is determined by5
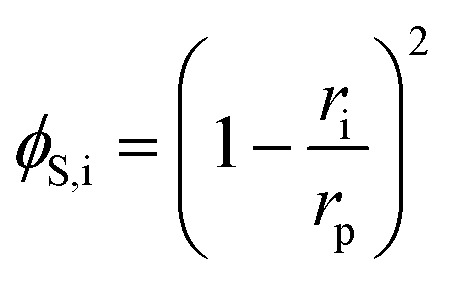
where *ϕ*_S,i_ is the steric exclusion term, *r*_i_ is the ion radius, and *r*_p_ is the pore radius. Donnan exclusion is defined by6
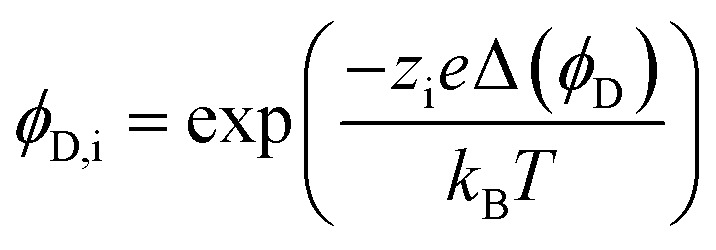
where *ϕ*_D,i_ is the Donnan exclusion term, *z*_i_ is ion charge, *e* is elemental charge, Δ(*ϕ*_D_) is the Donnan potential difference across the membrane from the feed to the permeate, *k*_B_ is the Boltzmann constant, and *T* is temperature. Dielectric exclusion is calculated by7
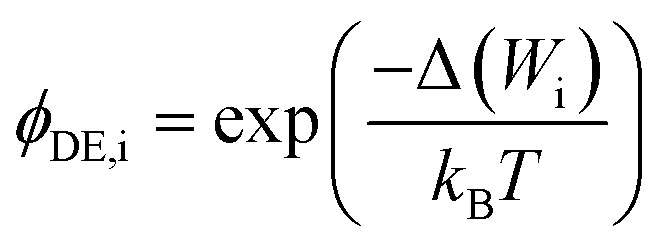
where *ϕ*_DE,i_ is the dielectric exclusion term and Δ(*W*_i_) is the solvation energy. The solvation energy can be calculated using the Born model:8
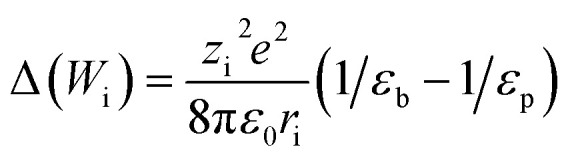
where *ε*_0_ is the dielectric constant of vacuum, *ε*_b_ is the dielectric constant of bulk solvent, and *ε*_p_ is the dielectric constant of solvent within the membrane.

Once the ion concentration at the membrane surface is known, an extended Nernst–Planck equation is used to calculate ion flux through the membrane, shown in eqn (9). Here, each term represents a distinct transport pathway: diffusion (first term), convection (second term), and electromigration (third term):9
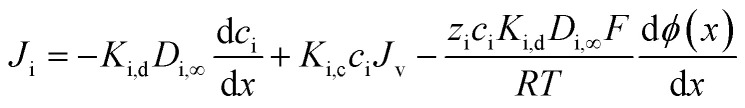
where *D*_i,∞_ is the diffusion coefficient of species i in the bulk solvent, *J*_v_ is the solvent flux, *F* is Faraday's constant, *R* is the ideal gas constant, and *ϕ*(*x*) is the potential at a point, *x*, across the membrane. Diffusive and convective hindrance factors, *K*_i,d_ and *K*_i,c_, account for friction between solutes and the pore walls but are defined separately for diffusive and convective transport.^27,53^

Despite more parameters, rejection mechanisms, and transport pathways than the SD model, the DSPM-DE still relies on several assumptions that limit its utility. These assumptions are reviewed in detail by Wang *et al.* but briefly, this model assumes uniform, straight, cylindrical and static pores, uniform membrane charge density, uniform membrane dielectric constant, spherical ions with Stokes radius, ideal solutions with negligible interactions, electroneutrality throughout the membrane, and one dimensional transport through the membrane.^53^ The additional parameters in the DSPM-DE model can be calculated experimentally but, even in a system that has been rigorously defined, the predictive capacity of the DSPM-DE model for different solutes, concentrations, and operating conditions is limited.

## 5 Knowledge gaps

Given the limitations of the current transport models, noted above, we identify knowledge gaps and motivate research to fill these gaps. The poor predictive capabilities of current models is attributed to a lack of membrane- and solute-specific parameters in transport equations and a poor of understanding of how polymer, membrane, and ion properties and interactions vary with environmental conditions.^65^Fig. 8a outlines the assumptions of the SD model and DSPM-DE while Fig. 8b illustrates how we may view ion transport as we move toward a molecular-level description. Below, we highlight specific knowledge gaps that, if filled, can improve our ability to understand and predict transport.

### 5.1 Membrane properties

Assumptions regarding membrane properties do not capture their heterogeneous and dynamic structure. Analytic models are based on whether a membrane is porous or dense, but the actual membrane structure probably has both dense and porous attributes, as discussed in Section 2.^105^ The SD model describes the membrane based on empirical parameters associated with partitioning and diffusion and even the more detailed DSPM-DE treats the membrane as a uniform material with constant thickness, charge distribution, and dielectric constant along with uniform, parallel, cylindrical pores. In reality, PA and other membranes form a highly heterogeneous surface in terms of morphology (ridge and valley structure, dynamic polymer chains with fluctuating voids) and chemistry (distribution of fixed charge groups).^106^

Current models assume one dimensional transport, but given the heterogeneous morphology of PA-RO membranes and variations in void size/shape, transport occurs through tortuous, three-dimensional pathways.^33^ Solutes likely follow lowest resistance pathways determined by local inhomogeneities in the membrane.^33^ The DSPM-DE attempts to account for additional transport resistances through hindered diffusion/convection coefficients, *K*_i,d_ and *K*_i,c_. These terms are meant to correct for friction between the solute and pore walls and are obtained using fitted, phenomenological equations based on the ratio of solute to pore radius.^53^ However, estimating the radii of ions in solution and the nature (size and shape) of PA voids is challenging and these terms do not account for ion–membrane interactions that can pose additional resistances.

### 5.2 Membrane surface *versus* interior

To evaluate the significance of interfacial partitioning and intrapore diffusion to overall transport, it is important to understand the properties and interactions at the membrane surface and interior. Ions and water molecules may experience opposing transport trends at the surface and in the interior of the membrane because the same interactions that draw ions into the membrane will slow their diffusion through it. For example, the large monovalent cation, Cs^+^, was shown to adsorb more favorably to the membrane surface but diffuse slower across the membrane interior compared to small, tightly hydrated ions.^60^ In the case of water transport, an increased density of hydrophilic groups at the membrane surface creates favorable interactions with water molecules, thereby reducing transport resistance at the interface. However, hydrophilicity in the membrane's interior slows water transport due to increased resistance from attractive interactions between water molecules and functional groups.^77^

The relative importance of ion partitioning *versus* diffusion has been found to vary for membranes with different properties; for example, studies on IX membranes have found that ion partitioning at the surface poses the dominant transport resistance compared to diffusion due to the large desolvation energy requirement and strong Donnan effects in IX polymers.^60,107^ In contrast, diffusion through the membrane's interior was found to create the largest transport resistance for RO membranes.^59,108^ There is still much to learn about membrane structure and chemistry, interactions, and transport and how these factors differ at the membrane surface and in the interior.

### 5.3 Water and the membrane

The state of water at the membrane surface and within the interior significantly affects ion and water permeability. Current assessments of membrane hydration lack the molecular details to understand and predict the implications of the state of water on the interactions of water and ions with the polymer and the consequent transport. Water's tendency to orient around charged groups reduces the electrostatic and dispersion energies between ions and membrane functionalities that encourage ions to partition into the membrane and create resistances that slow ion transport.^86^ An accurate determination of water's dielectric constant in the membrane is a prerequisite to properly account for the hydration and screening of membrane fixed charges and ions. Additionally, the degree of water swelling likely varies through the membrane thickness due to functional group concentration and confinement changes and probably fluctuates due to nanoscale heterogeneity and polymer dynamics.^33^ Given the importance of water content for transport kinetics, nonuniform swelling could increase the tortuosity of water/ion flow paths and affect the relative importance of partitioning and diffusion to overall transport.

### 5.4 Salt and the membrane

A better understanding of ion–membrane interactions and their implications on the transport mechanisms that govern membrane permselectivity is essential for predicting performance and for guiding development of more selective polymers. We presently lack a molecular level understanding of the hydration state of ions within a polymer membrane, how these partially hydrated ions interact with membrane functionalities, and how these phenomena depend on solution properties and membrane chemistry and morphology. This prevents prediction of ion sorption and transport.

In addition, the identity and concentration of ions that a membrane is exposed to impacts the membrane's properties, affecting interactions and transport behaviour noted above. As discussed in Sections 2 and 3, ion accumulation at the surface is dependent on salt concentration and varies for different ions. The build-up of ions at the membrane changes driving forces for ion and water transport and causes charge screening and structural changes, like deswelling, that alter the strength of subsequent interactions. Note that while high ion concentrations increase charge screening and deswelling, ion–membrane interactions at low concentration can be strong in the absence of significant charge screening and may also pose significant implications for transport.^65^

### 5.5 Ion and water coupled transport

Water molecules and ions likely traverse the membrane coupled together to some degree, however the extent of this coupled transport and its dependence on polymer membrane properties and ion identity is largely unknown. The SD model assumes that water and solute transport are completely independent and, while the DSPM-DE accounts for coupled transport of solutes and solvent in the convective transport term, ion and water transport may be more intertwined than the DSPM-DE considers.^105^

Solute uptake and mobility in the membrane has been shown to depend on the presence of bulk-like solvent in the membrane and properties like solvent viscosity.^109^ Water molecules can also transport coupled with ions *via* vehicular transport and favourable ion–membrane interactions may enhance this.^110^ In highly water-swollen (IX) membranes, coupled transport between ions and water is especially prominent.

In both the SD model and DSPM-DE, it is necessary to distinguish between the solute and solvent, which can be difficult in systems utilizing organic solvents or solvent mixtures and may not accurately represent transport when solute(s) and solvent(s) are highly coupled.^105^ A better understanding of the interplay between solute and solvent transport is critical to elucidating the molecular mechanisms of transport.

### 5.6 Complex solutions

Current models do not account for the impact of concentration or mixed-salt solutions on ion transport, despite evidence that this is important. Salt concentration has been found to significantly affect permeability;^111^ however, the SD model assumes a constant salt permeability coefficient and the DSPM-DE parameters are independent of concentration.^63,67^ Studies have only considered single salt solutions and models currently treat solute transport as independent from the transport of other solutes. One study has shown that NaCl rejection by NF membranes decreases in the presence of MgCl_2_ (ref. 112) but very little work has been done on the selectivity of solutes in multicomponent mixtures. To apply knowledge gained from molecular interactions to practical membrane design and operation, we need to develop an understanding of how the concentration and presence of different species affects interactions and transport.

### 5.7 Electroneutrality

Electroneutrality (compensating cation and anion concentrations) is a fundamental assumption of all current transport models but recent studies have shown that this assumption may break down locally in nanoporous systems.^82,113^ When a membrane carries a charge, complete screening is not possible inside nanoconfined channels and a potential difference develops between the inside and outside of that pore. Even for neutral membranes, a potential difference may develop based on hydration and non-electrostatic interfacial interactions.^82^ However, it is unclear if this effect applies to PA-RO membranes since a study on battery electrodes found that closely spaced pores may counteract local electroneutrality breakdown effects in nanopores and, instead, contribute to a potential difference through the electrode thickness.^113^ While the proposed void size of PA-RO membranes suggests that local loss of electroneutrality are possible, the dynamic nature of voids, uncertainty of internal charge based on the protonation of functional groups, and the variation in feed water composition makes the implications of electroneutrality on transport unclear. Given the prevailing assumption that electroneutrality is maintained throughout the system, it is intriguing to consider if local electroneutrality breakdowns influence nanoscale interactions.

## 6 A path to understand and leverage molecular level interactions

Above, we have reviewed what is known about the properties of hydrated ions and membranes, explored potential nanoscale interactions, and identified knowledge gaps that limit predictive capabilities of transport models and hinder the development of new membrane materials. Before we can design tunable materials and develop more sophisticated and predictive transport expressions, we must determine which ion/membrane properties and interactions are significant in dictating the overall permeability and selectivity. A multi-modal approach using a range of advanced characterization, electrochemical, and computational techniques is likely required to obtain a fundamental understanding of (1) the membrane structure and chemistry in the presence of water and ionic solutions, (2) the state of different cations/anions at the membrane surface and interior, and (3) the relationship between ion/membrane properties, interactions, and transport.

First and foremost, it is necessary that we accurately characterize the membrane structure in realistic hydrated, electrolytic, and pressurized environments. Elucidating the nature of voids in PA membranes and verifying the structure and chemistry of surface and interior membrane regions with *in situ*, depth-sensitive imaging, scattering, and spectroscopy characterization techniques would enable us to better model and visualize interactions in the membrane.^33^ Polymer chain dynamics can be difficult to probe but would give valuable information about the nature of voids and transport resistances in water and electrolyte solutions. Dielectric relaxation spectroscopy may help determine the nature of water in the membrane phase which would improve our ability to understand and model solvation states and subsequent interactions in the hydrated membrane.^75^

Secondly, it is imperative to improve our understanding of the hydration and bonding environments of ions in the membrane. The degree of ion solvation within the confined membrane phase must be determined, and we need to understand which ion–membrane interactions occur under various conditions. Advanced characterization techniques give the opportunity for *in situ*, spatial/temporal characterization of the local ion environment. For example, X-ray absorption spectroscopy (XAS)^114,115^ can be used to investigate ion–polymer interactions, the extent of ion hydration within a membrane and Ca-bridging interactions leading to fouling layer formation. Small and wide angle X-ray scattering^39,116–119^ probe porosity (void size and density) and local bonding motifs within the polymer membrane, respectively. Imaging methods (electron and X-ray microscopes)^33,120–122^ can be used to map heterogeneities in both composition, chemistry/bonding, and morphology/structure. From the polymer chain dynamics perspective, established methods such as quasi-elastic neutron scattering (QENS)^123^ and emerging methods including X-ray Photon Correlation Spectroscopy (XPCS)^124^ have considerable potential to provide insight into this understudied property and its relationship to free volume and transport. Note that many of these methods can be used in realistic operating environments relevant to RO and IX. For more details, we refer readers to a review on the application of advanced characterization to membranes by Bone *et al.*^12^

Molecular dynamics (MD) simulations and density functional theory (DFT) calculations can be used to predict polymer membrane structure, hydration, the interactions of ions and water with the membrane, and water and ion transport properties as summarized in ref. 125 and 126. These computational approaches allow construction of atomistic models of PA structure that can be directly compared to experimental characterization such as those noted above.^127^ MD studies of hydration show significant heterogeneity and water filling of permanent and dynamic voids, suggesting a dual porosity of PA with small and large pores.^128^ Most water (>90%) forms percolating dynamic, but highly tortuous, networks spanning the membrane. At a molecular scale, simulations of the permeation of water through the PA indicate a dynamic behavior of water molecules undergoing jump and other diffusion processes.^129^ MD simulations suggest that different ions lose their solvation shells to differing extents and some ions show preferential coordination to polymer functional groups,^126^ which highlights the importance of solvation shell loss in permselectivity. MD and DFT, properly vetted by experiments,^130^ can provide remarkable molecular insight into ion and solvent interactions and to transport mechanisms at an atom resolved level that is nearly impossible to obtain experimentally.^21,92,131,132^ These computational approaches can also greatly aid in the interpretation of characterization techniques noted above.

To holistically understand transport, we must be able to distinguish the unique interactions of cations from those of anions and differentiate between interactions at the membrane surface and within the membrane interior. Electrochemical impedance spectroscopy (EIS) can be used to deconvolute energy barriers associated with surface partitioning and interior diffusion components of transport by creating an equivalent circuit model to describe the system. EIS methods can also be used to distinguish cation and anion transport, to determine the extent of ion pairing, and to calculate individual energy barriers.^59^ The ability to perform real time, *in situ* experiments make EIS highly complementary to advanced structural and chemical characterization and simulations. The theory and principles of impedance studies have been established by Freger and Bason,^133^ and recent studies have demonstrated the unique insight on ion permeation and membrane performance that can be gained from such techniques.^108,134–136^

Once dominant interactions are identified and their impact on energy barriers for partitioning and diffusion are understood, membrane performance measurements will allow us to systematically determine the relationship between molecular-scale interactions and membrane functionality. Ion and water permeability measurements can be gathered in a variety of operating conditions—from bench-scale filtration cells to full size, spiral-wound membrane modules—to explore the effect of forces at different size scales on transport. A particular emphasis should be placed on studying a variety of cations and anions and mixed-electrolyte solutions. The behaviour of NaCl cannot be used to predict the behaviour of other solutes and the transport of a solute is affected by other components in solution. A public database for experimental and computational results under a wide variety of conditions, such as the one created by Ritt *et al.*, would be helpful to streamline research efforts and allow for statistical analysis and machine learning to reveal trends.^137,138^ This is also a good opportunity to use molecular dynamics simulations to systematically study the effects of different ion/membrane properties and environmental conditions on interactions and transport.

## 7 RO in the context of sustainability and social justice

Advancing RO technology requires understanding the environmental and social contexts in which it is implemented. From an environmental perspective, it is clear that seawater desalination, even by RO, is energy intensive and the use of RO to produce water should be considered in the context of less energy-intensive solutions that would reduce our need to tap into seawater sources, including wastewater reuse and more water-efficient technologies. To ensure an adequate clean water supply, we must work to create a more sustainable relationship with water by protecting existing freshwater sources and utilizing them responsibly. First, we can increase harvesting of less resource intensive water sources through practices like rainwater collection, increased green spaces to retain rainwater, infrastructure repair, and improved catchment and distribution systems.^139,140^ Wastewater reuse also offers a lower energy water supply and desalination with limited extraction from the environment, and RO can be a valuable technology for this.^5^ Second, water usage can be greatly reduced in agriculture, industry, and residential homes/businesses by eliminating unnecessary use and by reusing lightly-used grey water for applications that do not require potable or clean water.^141^ Finally, it is critical that we protect the quality of our water sources from known and yet unidentified chemicals that pose significant threats to environmental and human health. These protections require more proactive and comprehensive regulations on industrial/agricultural discharge.

One way public health is prioritized is through the precautionary principle whereby companies must prove the safety of chemicals before production and distribution. This principle is implemented in many countries but not the U.S., having far-reaching impacts on the environment and public health.^142–144^ The impact of RO membranes on the environment should be considered since membranes are synthesized using toxic solvents and reagents that could potentially be replaced with less hazardous alternatives.^145^ Additionally, RO systems produce a potentially hazardous brine stream that must be safely treated and discharged or ideally reused.^146^

While a multi-faceted approach will improve our capacity to generate and utilize water resources more sustainably, we must keep in mind that technical developments are not the only methods to address challenges of water quantity and quality. More aggressive water conservation policies and increased awareness/adoption of conscientious water practices will also be necessary for developing a sustainable relationship with our water supply.

Importantly, water scarcity occurs in the context of numerous political and social forces and technological advancements do not always translate into better quality of life, especially for those who need such improvements most.^147,148^ The World Health Organization recognizes that access to clean water is critical to poverty reduction and economic prosperity.^149^ Engineers and scientists can work to make technologies more accessible to low-income populations, but much of the change must happen through social activism and policy reform, which can be informed by science.^144^ Research goals should specifically work to expose and address water challenges that affect marginalized populations since water contamination from industrial runoff and inadequate sanitation infrastructure is most prominent in these communities.^150,151^

An example of framing goals in this manner is in the area of lead and perfluoroalkyl substances (PFAS) contamination where, in addition to developing technologies to remove toxic compounds, scientists have worked across disciplines to highlight disparities in exposure to these harmful chemicals and link them to environmental and human health outcomes.^152–155^ Successful consideration of how water challenges affect communities requires thoughtfully communicating findings to inform the public and building an understanding of concerns from communities with diverse political, cultural, religious, ethnic, and environmental perspectives.^156^ Finally, we must consider how the structure of colonialism encourages inequalities to persist and think critically about how scientific advancement changes society in unintended ways so that we can work toward a more habitable world for all.^157^

## 8 Outlook

With water security at the forefront of the challenges faced by humanity in coming years, better membrane materials for purifying water from a variety of sources are essential. More specifically-tuned separation materials will also be essential for batteries, pharmaceuticals, carbon dioxide capture, and contaminant cleanup. To create these necessary technologies, we must develop a better molecular understanding of the mechanisms that govern the performance of current materials, including polyamide for RO membranes, which will enable design of novel materials.

Elucidating nanoscale interactions and linking them to membrane/ion properties and performance over a range of environmental conditions will allow the field to better understand and leverage the mechanisms that drive permeability and selectivity performance. The insights gained will allow for determination of more realistic transport/exclusion mechanisms and more accurate ion- and membrane-specific parameters that are used in performance models. These findings can then be used to modify current models and inform membrane material and system design to facilitate desired interactions and improve membrane performance in a range of applications. The techniques and experimental procedures developed in pursuit of this knowledge will also be applicable to important technologies in clean energy, water, biological, and medical fields that commonly depend on ion interactions with soft matter. While it has previously been difficult to obtain direct realistic measurements of the properties and interactions of hydrated ions and polymers, recent advances in *in situ* characterization, electrochemical, and computational methods could enable these insights, especially when closely linked together.

## Data availability

Since this is a review paper, all data is contained in the relevent referenced papers.

## Author contributions

All the authors conceived of this Perspective, discussed the concepts, and commented on the manuscript. T. R. N. wrote the first draft and all authors edited the subsequent drafts.

## Conflicts of interest

There are no conflicts to declare.

## Supplementary Material
